# A Systematic Review of *Orthosiphon stamineus* Benth. in the Treatment of Diabetes and Its Complications

**DOI:** 10.3390/molecules27020444

**Published:** 2022-01-10

**Authors:** Qirou Wang, Jia Wang, Nannan Li, Junyu Liu, Jingna Zhou, Pengwei Zhuang, Haixia Chen

**Affiliations:** 1 Tianjin Key Laboratory for Modern Drug Delivery & High-Efficiency, School of Pharmaceutical Science and Technology, Tianjin University, Tianjin 300072, China; wangqirou110@163.com (Q.W.); jiawangcongcong@163.com (J.W.); 13234039725@163.com (N.L.); junyuliu@tju.edu.cn (J.L.); jingnazhou66@163.com (J.Z.); 2 Haihe Laboratory of Modern Chinese Medicine, Chinese Materia Medica College, Tianjin University of Traditional Chinese Medicine, Tianjin 301617, China; zhuangpengwei@163.com

**Keywords:** *Orthosiphon stamineus*, diabetes, diabetic complications, hypoglycemic activity, antidiabetic mechanisms

## Abstract

(1) Background: *Orthosiphon stamineus* Benth. is a traditional medicine used in the treatment of diabetes and chronic renal failure in southern China, Malaysia, and Thailand. Diabetes is a chronic metabolic disease and the number of diabetic patients in the world is increasing. This review aimed to systematically review the effects of *O. stamineus* in the treatment of diabetes and its complications and the pharmacodynamic material basis. (2) Methods: This systematic review was conducted following Preferred Reporting Items for Systematic Reviews and Meta-Analyses (PRISMA), using the databases ScienceDirect, PubMed, and Web of Science. (3) Results: Thirty-one articles related to *O. stamineus* and diabetes were included. The mechanisms of *O. stamineus* in the treatment of diabetes and its complications mainly included inhibiting α-amylase and α-glucosidase activities, antioxidant and anti-inflammatory activities, regulating lipid metabolism, promoting insulin secretion, ameliorating insulin resistance, increasing glucose uptake, promoting glycolysis, inhibiting gluconeogenesis, promoting glucagon-likepeptide-1 (GLP-1) secretion and antiglycation activity. Phenolic acids, flavonoids and triterpenoids might be the main components for hypoglycemia effects in *O. stamineus*. (4) Conclusion: *O. stamineus* could be an antidiabetic agent to treat diabetes and its complications. However, it needs further study on a pharmacodynamic substance basis and the mechanisms of effective constituents.

## 1. Introduction

*Orthosiphon stamineus* Benth. (Lamiaceae) is a perennial herb [1,2]. *O. stamineus* is widely distributed in the tropical and subtropical regions [3], including southeast Asian countries (Indonesia, Malaysia, Thailand, Vietnam, Myanmar, Philippines) [4,5], southern China [6], India [7], Australia [5], etc. In addition to *Orthosiphon stamineus* Benth., it also has other scientific names, *Clerodendranthus spicatus* (Thunb) c. y. wu and *Orthosiphon aristatus* (Blume) Miq. [8,9,10]. It is usually called “Shencha” in Chinese. It is also called Cat’s whiskers [11], Misai Kucing [12], Java tea [13], and kumis kucing [14] in some Southeast Asian countries.

*O. stamineus* is a popular Chinese folk medicine and also a traditional medicine of Dai nationality of Yunnan Province in China [15]. It has been used to treat diabetes and some kidney diseases with a long history. Modern pharmacological studies show that *O. stamineus* has many pharmacological activities, including antioxidant, anti-inflammatory, kidney protection, antibacterial, anti-tumor, immunoregulation, and especially effective antidiabetic activities. [15,16]. It has been used for the treatment of diabetes and chronic renal failure clinically. It is also reported to have good therapeutic effects on some diabetic complications, especially diabetic nephropathy [6]. Thus, it is worthy of study for the discovery for new antidiabetic drugs from *O. stamineus*.

Diabetes is a chronic metabolic diseases caused by deficiency in insulin secretion and insulin resistance [17]. In 2021, diabetic patients were estimated to be approximately 537 million all over the world [18]. This number is on the rise, the reasons for which are an aging population, obesity, and unhealthy diets [19]. Diabetes can be classified into two major types: Type I Diabetes Mellitus (T1DM) and Type II Diabetes Mellitus (T2DM). T1DM is caused by insulin deficiency. The islet β-cells are damaged, leading to an absolute deficiency of insulin secretion. Patients need long-term exogenous insulin injection. However, for T2DM patients, metabolic disorder results in lower insulin sensitivity, insulin resistance, and relative insulin deficiency [17,19,20,21]. DM can damage organs and tissues and result in many complications, such as diabetic nephropathy, diabetic retinopathy, diabetic foot, diabetic neuropathy, etc.

Diabetes is treated with oral hypoglycemic drugs and insulin injection to reduce blood glucose levels, improve insulin secretion, and enhance insulin sensitivity. Besides, there also are natural products used in the treatment of diabetes, especially with good hypoglycemic effects. In classical antidiabetic drugs, exenatide is from the venom of Gila monster and acarbose is produced from *Actinoplanes* sp. by the large-scale fermentation [22,23]. Besides, metformin is a natural product derivative that originated from herbal medicine *Galega officinalis* and its constituent galegine [24]. Many other natural products, such as curcumin, cinnamon, pumpkin, bitter melon, *Lycium barbarum*, *Portulaca oleracea*, *Aloe vera*, etc., have also been proven to have antidiabetic activities but without general clinical practice [20,21,25,26,27]. *Orthosiphon stamineus* also has potential against diabetes.

More than 200 compounds have been isolated from *O. stamineus*. Diterpenoids, flavonoids, triterpenoids, phenolic acids, and their derivatives are the main chemical constituents. There are almost 50 phenolic acids and their derivatives isolated from *O. stamineus*, including rosmarinic acid, caffeic acid, and their derivatives, and many others [6,28]. More than 20 flavonoids have been isolated from *O. stamineus*. Most of them are flavones, especially polymethoxy substituted flavones [29]. Besides, more than 60 diterpenoids have been isolated up to now and they have various skeleton types, including isopimarane [30], staminane [31], secoisopimarane [30], norstaminane [31], secostaminane, and some other types. Besides, there are also almost 20 triterpenoids isolated from *O. stamineus* [5,32,33]. In addition to the above four main types, there are also other kinds of compounds isolated from *O. stamineus*, such as two alkyl glycosides (clerspides A and B) [34], coumarins, etc. Many of the compounds studied have acted as the main pharmacodynamic material basis of *O. stamineus* in the treatment of diabetes and its complications.

In a review by Omar Z. Ameer, the traditional uses, phytochemical studies, pharmacological studies, and toxicology were summarized. In Kamran Ashraf’s review, only phytochemical studies and pharmacological studies until 2018 were summarized. The pharmacological activities of *O. stamineus* mentioned in the two reviews included anti-inflammatory, antioxidant, diuretic, hypouricemic, hepatoprotective, gastroprotective, nephroprotective, analgesic, antipyretic, cytotoxic, antiproliferative, antihypertensive, hypoglycemic, hypolipidemic, anti-obesity, and antibacterial activities [35,36]. In Yin-Sir Chung’s review, the protective actions of *O. stamineus* on the central nervous system, safety and toxicity, and pharmacokinetics studies were summarized [8]. Although there are many studies on the potential of *O. stamineus* in the treatment of diabetes and its complications, no article has reviewed the antidiabetic mechanisms and pharmacodynamic material basis of *O. stamineus* in detail.

In this systematic review, the mechanisms and toxicology of *O. stamineus* in the treatment of diabetes and its complications were summarized as per Preferred Reporting Items for Systematic Reviews and Meta-Analyses (PRISMA) [37,38]. PRISMA provides guidance for authors to prepare transparent, complete, and accurate systematic reviews. Research progress on clinical applications and the main pharmacodynamic material basis of *O. stamineus* was reviewed, providing a reference for the application of *O. stamineus* and further research in the treatment of diabetes and its complications.

## 2. Results

### 2.1. Literature Search Results

**Figure 1:**
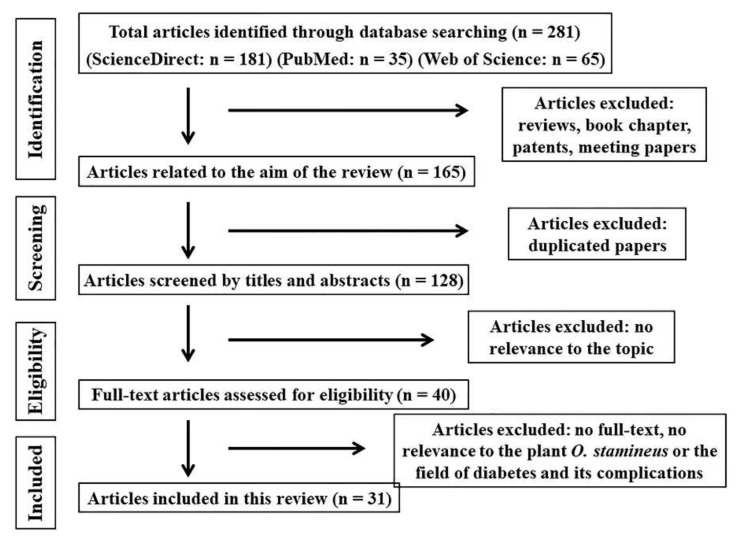
The flowchart of the literature search and selection process based on PRISMA.

After searching in the three databases by using the chosen keywords, a total of 281 studies were obtained. Of the 281 records, 181 were from ScienceDirect, 35 from PubMed, and 65 from Web of Science. Then, 153 records were removed for the following reasons: duplicate studies, reviews, book chapters, patents, meeting papers, and non-English language papers. By reviewing the titles and abstracts, 88 records were excluded because they had no relevance to the scope of this review. The remaining 40 records were read fully, and 31 were included in this systematic review. The flowchart of the literature search and selection process is shown in Figure 1 and the 31 articles are summarized in Table 1.

### 2.2. Hypoglycemic Activity

Hyperglycemia is a main symptom of diabetes, and could cause damage to organs and tissues in the body. It has been proved in some studies that different extracts of *O. stamineus* could decrease blood glucose levels.

In a recent study, the 95% ethanol elution fraction (95% EEF) of 80% ethanol extract (0.68 g/kg, 0.34 g/kg and 0.17 g/kg) reduced blood glucose levels in an oral glucose tolerance test in normal C57BL/6J mice after 10-day administration of the extract [39]. The ethanol extract of *O. stamineus* (0.2 and 0.4 g/kg) obviously reduced fasting blood glucose level in high-fat-diet (HFD) C57BL/6 mice after 8-week administration of the extract [40]. The rats were administered 50% ethanol extract orally and after ten minutes, they were loaded with starch or sucrose. The extract (1 g/kg) reduced blood glucose levels significantly after starch loading in both normal and diabetic rats. The same dose of the extract also lowered blood glucose levels significantly after sucrose loading in normal rats [41]. The rats were administered chloroform extract and its sub-fraction 2 (1 g/kg) orally and after one hour, they were loaded subcutaneously with glucose. The extract and its sub-fraction 2 significantly reduced the blood glucose levels of normal rats [42]. The same sub-fraction (1 g/kg) also caused a significant decrease in blood glucose levels in diabetic rats after 14-day administration of the sub-fraction [34]. The normal and diabetic rats were administered the aqueous extract orally and after ten minutes, they were loaded with glucose. In normal rats, the aqueous extract (0.5 g/kg and 1.0 g/kg) reduced plasma glucose concentration by 15% and 34%, respectively, after one hour of glucose loading. The maximum reduction of the extract (0.5 g/kg and 1.0 g/kg) on diabetic rats was 21% and 24% after 210 min of glucose loading. Besides, the diabetic rats were also treated with the extract (0.5 g/kg) for 14 days and showed reduction in plasma glucose concentration [43].

### 2.3. Mechanisms of O. stamineus in the Treatment of Diabetes

#### 2.3.1. Antioxidant Activity

Hyperglycemia metabolism and excessive free fatty acids can lead to the production of lots of free radicals, such as reactive oxygen species (ROS) and reactive nitrogen species (RNS). These free radicals can cause oxidative stress, impair the structures and functions of islet β-cells, and cause insulin secretion deficiency. Besides, they can also lead to insulin resistance by affecting multiple insulin signaling pathways. The antioxidant activity of *O. stamineus* is related to protecting islet cells and reducing insulin resistance. Researchers have always tested antioxidant activity by 1,1-diphenyl-2-picrylhydrazyl radical 2,2-diphenyl-1-(2,4,6-trinitrophenyl)hydrazyl (DPPH) assay, ferric ion reducing antioxidant power (FRAP) assay, and 2,2′-azino-bis-(3-ethylbenzothiazoline-6-sulphonate) (ABTS) assay. The activity of superoxide dismutase (SOD) and the level of malondialdehyde (MDA) are also used to determine antioxidant activities. SOD can scavenge free radicals and MDA is the end product of lipid oxidation [44,45].

The antioxidant properties of the ethanol extracts of some genotypes ranged up to 15.55 μmol trolox equivalent (TE)/g dry weight (DW) in DPPH assay, and ranged up to 1.60 mmol TE/g DW in FRAP assay [46]. The half maximal inhibitory concentration (IC50) value of the 70% ethanol extract was 58.85 ± 7.11 μg/mL in DPPH assay, a little higher than 15.05 ± 2.03 μg/mL of the positive control, rosmarinic acid [47]. The concentration value for 50% of maximal effect (EC50) of methanol extract was 0.67 mg/mL in DPPH assay [48]. The IC50 values of 50% methanol extract of *O. stamineus* leaves were 0.145 ± 0.030, 1.143 ± 0.056, 0.192 ± 0.012, and 0.013 ± 0.001 mg/mL in DPPH, ABTS, iron chelating and FRAP assays, respectively, a little higher than the positive control, rutin and caffeic acid [49]. The EC50 values of *O. stamineus* aqueous extract were 53.51 and 284.9 μg/mL, respectively, in DPPH and ABTS assays, higher than the positive control, ascorbic acid [50]. The antioxidant capacities of aqueous extract were higher than 20 mg ascorbic acid equivalents (VCEAC)/100 mL in ABTS assays, and about 40 mg VCEAC/100 mL in FRAP assays [51]. The DPPH free radical-scavenging activities of aqueous, 50% methanol, methanol, 70% acetone, and chloroform extracts (0.05 mg/mL) were about 85%, 90%, 88%, 83% and 70%, respectively, higher than some positive controls [52].

From these studies, it could be seen that the aqueous extract, ethanol extract, 70% ethanol extract, methanol extract, 50% methanol extract, 70% acetone extract, and chloroform extract all had free radical-scavenging activities in different assays.

*O. stamineus* ethanol extract (200 and 400 mg/kg) enhanced SOD activity and reduced MDA level in the liver homogenate of the high-fat diet group. Thus, *O. stamineus* extract might counteract oxidative stress in the liver [40]. The 50% ethanol extracts of *O. stamineus* roots, stems, and leaves (50 μg/mL) scavenged intracellular ROS and significantly increased cell viability under oxidative stress in IPEC-J2 cells. They could also decrease the MDA level in jejunal homogenates compared to the high-fat group. The extracts of roots and leaves significantly increased the jejunal SOD activity of mice [53].

#### 2.3.2. Anti-Inflammatory Activity

In the pathogenesis of diabetes, inflammatory factors, such as interleukin (IL)-1β, IL-8, tumor necrosis factor (TNF)-α, and induced nitric oxide synthase (iNOS), are important factors related to insulin sensitivity. They interfere with insulin signal transduction by participating in the insulin signaling pathway, leading to insulin resistance. They also possibly damage islet β-cells. In addition, inflammatory factors also interact with oxidative stress, further aggravating insulin resistance. Therefore, anti-inflammatory activity is essential to attenuate the inflammatory response, protect islet cells, and improve insulin resistance. It is always tested through the levels of inflammatory factors and the inhibition of nitric oxide (NO) production in cells [54,55].

The swelling in auricle was inhibited by the treatment of ethanol extract, ethyl acetate (EtOAc), and aqueous fractions in acute inflammatory mice induced by xylene. The inhibition ratios were 48.2%, 63.3%, and 46.0% at the dose of 200 mg/kg. Some compounds isolated from EtOAc fractions, orthosiphol M, orthosiphonone A, orthosiphol B, neoorthosiphol A, orthosiphol D, fragransin B_1_, sinensetin and 5, 6, 7, 4′-tetramethoxyflavone, also showed marked repression in the observed auricle swelling at the dose of 50 mg/kg. Besides, some of these compounds inhibited pro-inflammatory cytokines production in lipopolysaccharide (LPS)-induced HK-2 cells, such as the levels of TNF-α, IL-1β, and IL-8 [56]. The isolated compounds (clerodens A–D) were studied for anti-inflammatory activities on LPS-induced NO production in RAW264.7 macrophages. The results showed that clerodens A–D had inhibitory activities with IC50 values of 18.9 ± 1.2, 14.7 ± 0.48, 12.4 ± 1.5, and 6.8 ± 0.92 μmol/L, respectively, a little higher than the positive control aminoguanidine [16]. Neoorthosiphonone A, isolated from *O. stamineus*, showed obvious inhibitory activity on NO production in LPS-activated macrophage-like J774.1 cells with the IC50 value of 7.08 μmol/L, which was more potent than the positive control N^G^-monomethyl-_L_-arginine (_L_-NMMA) [57]. The isolated siphonols A–E also inhibited NO production in LPS-activated macrophage-like J774.1 cells [58].

#### 2.3.3. Regulate Lipid Metabolism

Diabetic patients often have abnormal lipid metabolism. In the pathogenesis of diabetes, disorders in lipid metabolism increase the levels of free fatty acids and total triglycerides (TG), damaging islet β-cells and leading to insulin resistance in other tissue cells. Because of insulin resistance, the serum levels of TG, total cholesterol (TC), and low-density lipoprotein cholesterol (LDL-C) increase, while the level of high-density lipoprotein cholesterol (HDL-C) decreases [59]. In addition, leptin and adiponectin, which are secreted from adipocytes, are also associated with insulin resistance. Leptin can antagonize insulin and produce insulin resistance, while adiponectin can improve insulin sensitivity by increasing fatty acid oxidation and glucose uptake in skeletal muscle cells [60,61].

The inhibitory effect of *O. stamineus* ethanol extract against pancreatic lipase in vitro was determined by using orlistat as the positive control. The IC50 value of the extract was 5.7 mg/mL, compared to the value of orlistat (0.1 mg/mL). In vivo study, the mice were fed on HFD. The ethanol extract reduced the serum levels of TG, TC, LDL-C, and lipase. It also decreased the leptin level and increased the adiponectin level. The extract also attenuated excessive accumulation of fat in liver tissues through histological examination. These results all showed that the extract might regulate lipid metabolisms in adipocytes, downregulate lipid accumulation in the liver [40]. The aqueous extract lowered TC level and increased the ghrelin level in diabetic rats [62]. The aqueous extract also lowered TG level and increased HDL-C level in diabetic rats [43]. 3-Hydroxybutyrate (3-HBT) and acetoacetate were the representative metabolites of fatty acid metabolism, so their levels might be related to the lipid metabolism in the liver. In ^1^H-NMR spectroscopic analysis of urine of Azam’ study, aqueous extract showed a remarkable drop in acetoacetate and 3-HBT levels. The reason for that might be that the extract inhibited the abnormal lipid and fatty acid metabolism and re-established energy metabolism [63].

#### 2.3.4. Inhibit the Activities of α-Amylase and α-Glucosidase

α-Amylase and α-glucosidase are the two key enzymes in the digestion and absorption of carbohydrates in the body. α-Amylase breaks down long-chain carbohydrates, and α-glucosidase hydrolyzes glucoside bonds to release glucose. They are directly involved in the metabolism of starch and glycogen. Therefore, inhibiting the activities of α-amylase and α-glucosidase can reduce the release of glucose from carbohydrate hydrolysis, slow down the absorption of glucose in the small intestine, and effectively lower postprandial blood glucose level [44,64,65]. The inhibitory activities of these enzymes were always tested in vitro.

Rosmarinic acid and 2-caffeoyl-L-tartaric acid were two constituents isolated from *O. stamineus*. In a recent study, their inhibition ratios on α-glucosidase (0.5 U/mL) were 71.06 ± 1.82% and 69.85 ± 1.27%, respectively, both higher than that of positive control, acarbose, at concentration of 5 mg/mL. Molecular docking results showed that the binding energy of 2-caffeoyl-L-tartaric acid and α-glucosidase was −7.7 kcal/mol, and there were 3 hydrogen bonds between them. The binding energy of rosmarinic acid and α-glucosidase was −8.6 kcal/mol. In the conformation of α-glucosidase-rosmarinic acid complex, there were 6 hydrogen bonds [66]. The 95% EEF showed higher α-glucosidase (86 μg/mL) inhibitory activity (IC50 = 40 ± 0.73 μg/mL) than acarbose (IC50 = 250 ± 1.05 μg/mL) [39]. The ethanol extract (1000 μg/mL) of some genotypes of *O. stamineus* inhibited α-glucosidase up to 62.84% [46]. The ethanol extract at concentration of 50 μg/mL inhibited α-glucosidase (0.57 U/mL) at 40.74%, α-amylase (1.6 U/mL) at 81.48%, higher than acarbose[67]. The 50% ethanol extract and the isolated compound sinensetin both showed inhibitory activity on α-glucosidase and α-amylase. The IC50 values on α-glucosidase (1.0 U/mL) were 4.63 ± 0.413 and 0.66 ± 0.025 mg/mL, and on α-amylase (0.5 mg/mL) were 36.70 ± 0.546 and 1.13 ± 0.026 mg/mL, respectively. The IC50 values of acarbose on α-glucosidase and α-amylase were 1.93 ± 0.281 mg/mL and 4.89 ± 0.397 mg/mL, respectively [68].

#### 2.3.5. Promote Insulin Secretion, Ameliorate Insulin Resistance, Enhance Insulin Sensitivity

Insulin is a hormone secreted by islet β-cells. It can control blood glucose level and regulate glucose and lipid metabolism. Insulin promotes glucose uptake and utilization in the liver, muscle, and adipose cells to reduce postprandial blood glucose level. However, these functions can be achieved only by combining with insulin receptors (IR). IRs are widely distributed in the body. Muscle, fat, and liver are all insulin target organs or tissues. Insulin resistance occurs when insulin receptors become less sensitive to insulin due to various factors [69]. Normally, glucose is transported and utilized mainly under the stimulation of insulin through a variety of insulin signaling pathways, such as the phosphoinositide 3-kinase/protein kinase B (PI3k/Akt) pathway. Insulin binds to IRs on the cell membrane, causing tyrosine phosphorylation of insulin receptor substrates (IRS), activating the PI3k/Akt signaling pathway and increasing glucose uptake. Any abnormality in insulin signaling pathway may lead to insulin resistance [70,71]. In addition, protein tyrosine phosphatase 1B (PTP1B) is also associated with insulin resistance. High PTP1B activity can lead to the dephosphorylation of IR and IRS tyrosine and weaken insulin signal transduction, leading to insulin resistance [72,73]. In some investigations, it has been proved that the extract of *O. stamineus* and its active components promoted insulin secretion, improved insulin resistance, and enhanced insulin sensitivity.

Inhibition of PTP1B activity might improve IR and IRS, leading to the improvement of insulin resistance and enhancement of insulin sensitivity. Hence, five diterpenes isolated from *O. stamineus* were tested for PTP1B inhibitory activity. The IC50 values of siphonol B, orthosiphols B, G, I, and N were 8.18 ± 0.41, 9.84 ± 0.33, 3.82 ± 0.20, 0.33 ± 0.07, and 1.60 ± 0.17 μmol/L, respectively, compared to the positive control, ursolic acid (3.42 ± 0.26 μmol/L). The inhibition types of these five diterpenes on PTP1B were mixed-competitive, non-competitive, non-competitive, competitive, and uncompetitive, respectively [74]. The hexane fraction of 70% ethanol extract slightly increased insulin secretion in both basal and glucose-stimulated states, and also elevated the mRNA expression of insulin and pancreatic duodenal homeobox-1 (PDX-1) in INS-1 cells under normal and high-glucose conditions. PDX-1 is an essential transcription factor for insulin gene expression. Its main functions are to promote the proliferation of islet β-cells, inhibit the apoptosis of islet β-cells, and regulate the transcription of insulin genes. The fraction also increased p-PI3K levels and Akt phosphorylation in INS-1 cells [75]. The ethanol extract reduced the levels of homeostasis model assessment of insulin resistance (HOMA-IR) index in HFD-induced rats [40].

From these studies, it could be seen that the hexane fraction of 70% ethanol extract could promote insulin secretion and enhance insulin sensitivity. Besides, the ethanol extract and five diterpenes isolated from *O. stamineus* could both enhance insulin sensitivity.

#### 2.3.6. Reduce the Absorption of Intestinal Glucose, Increase Glucose Uptake by Peripheral Cells

Hyperglycemia is a typical characteristic of diabetes. Carbohydrates are absorbed by intestinal epithelial cells in the form of glucose after digestion by enzymes. The uptake and utilization of glucose mainly exist in peripheral tissues or cells, such as liver, muscle, and adipose cells. Therefore, reducing the absorption of intestinal glucose and promoting glucose uptake by peripheral cells are very important to reduce blood glucose [76].

The sub-fraction 2 of chloroform extract significantly inhibited the glucose absorption from the small intestine at concentrations of 0.5, 1.0, and 2.0 mg/mL. Sub-fraction 2 (2.0 mg/mL) significantly increased the glucose uptake of hemi-diaphragms during the 90-min incubation period [34]. Some diterpenes in *O. stamineus* had 2-deoxy-2-((7-nitro-2,1,3-benzoxadiazol-4-yl)amino)-D-glucose (2-NBDG) uptake effect in 3T3-L1 adipocytes. 2-NBDG was always used as a substrate to evaluate the action of compounds as insulin mimickers. Siphonol B, orthosiphols B, G, I, and N stimulated glucose uptake at the concentration of 5 and 10 μmol/L [74]. The aqueous extract of *O. stamineus* significantly enhanced glucose uptake and glucose consumption in 3T3-L1 adipocytes [77]. The *O. stamineus* aqueous extract could increase the glucose uptake in cells by measuring the traces of radiolabelled glucose in 3T3-L1 adipocytes model [78].

#### 2.3.7. Promote Glycolysis, Inhibit Gluconeogenesis

Gluconeogenesis and glycolysis are two metabolic mechanisms to ensure glucose homeostasis. Glycolysis is the process of breaking down glucose to produce pyruvate, which is one of the most important pathways of glucose metabolism in the body. Increasing the expression of glucokinase and pyruvate kinase can promote glycolysis and reduce blood glucose. Gluconeogenesis is the process of converting non-sugar substances into glucose. Liver is the main organ for gluconeogenesis. Both insulin and glucagon can regulate liver gluconeogenesis through different signaling pathways [79,80].

In ^1^H-NMR spectroscopic analysis of urine of diabetic rats, aqueous extract increased the levels of pyruvate, succinate, and citrate compared to the model group. Pyruvate is an end product of glycolysis, and it can enter tricarboxylic acid (TCA) cycle. High glucose level inhibits glycolytic enzymes and decreases the generation of pyruvate, thereby reducing the TCA cycle activity, and thus may contribute to mitochondrial dysfunction. Mitochondrial dysfunction may induce diabetes by affecting insulin secretion of islet β-cells and aggravating insulin resistance. Citrate and succinate are the TCA cycle intermediates. Thus, the increased levels of pyruvate, citrate, and succinate showed that the aqueous extract might reduce blood glucose level by increasing glycolysis and decreasing gluconeogenesis, and it might also modulate TCA cycle and improve mitochondrial dysfunction [63].

#### 2.3.8. Increase the Level of GLP-1

GLP-1 is released from intestinal cells and maintains blood glucose homeostasis by increasing insulin secretion and inhibiting glucagon secretion [81]. The aqueous extract of *O. stamineus* (0.1 g/100 g of body weight) increased GLP-1 level in diabetic rats—non-pregnant or pregnant [62].

### 2.4. Mechanisms of O. stamineus in the Treatment of Diabetic Complications

Chronic hyperglycemia may cause damage to vessels and microvessels, and also damage tissues and organs in the body, leading to diabetic nephropathy, diabetic retinopathy, diabetic foot, diabetic peripheral neuropathy, and diabetic cardiovascular complications. These diabetic complications are related to oxidative stress, nonenzymatic glycation of protein, and inflammatory factors [82].

In addition to antioxidant and anti-inflammatory activity, *O. stamineus* also has anti-glycation effects. The glycation process is the formation of Amadori products at first through the chemical reactions between amino acid residues in proteins and reducing sugars. These products transform into advanced glycation end products (AGEs) by dehydration and rearrangement reactions. The accumulation of AGEs is toxic to cells and tissues, leading to diabetic complications. The aqueous extract of *O. stamineus* had inhibitory capacities (more than 70%) on the formation of AGEs in bovine serum albumin (BSA)-glucose system [51].

Diabetic nephropathy (DN) is one of the main complications of diabetes. It may lead to renal failure. The *O. stamineus* aqueous extract lowered the 24 h urine albumin excretion rate (UAER), glomerular filtration rate (GFR), the index of kidney weight to body weight and MDA level in kidney tissues of diabetic rats. It also improved the activity of SOD in renal tissues. Under a light microscope, *O. stamineus* obviously improved the lesions of renal tissues. The protective effect of *O. stamineus* on diabetic rats may be related to antioxidative activity, anti-inflammatory activity, and inhibition of the proliferation of mesangial cells [83].

### 2.5. Toxicity

Even though most traditional herbal medicines are generally recognized as safe, they also need to be evaluated the safety and toxicity. Toxicology studies have led to a better understanding of human physiology and drug interactions with the body.

There was no cytotoxicity effect of *O. stamineus* aqueous extract on 1.1B4, 3T3-L1, and WRL-68 cells viability during 24 h treatment at a concentration of 1.0 mg/mL. In fish embryo acute toxicity (FET) test on zebrafish, there was also no mortality on zebrafish embryos at 1.0 mg/mL [50].

Several studies were about the possible toxicity of *O. stamineus* in rats. In an acute toxicity study, the aqueous, 50% ethanol and ethanol extracts of *O. stamineus* (5000 mg/kg) were administered orally to rats for 14 days. In other acute studies, methanol extract and 50% ethanol extract were also administered to rats. While in the subchronic toxicity study, the 50% ethanol extract was administered orally at doses of 1250, 2500, and 5000 mg/kg for 28 days. There was no mortality or any signs of toxicity during the experiment periods. There was also no significant difference in body weight, organ weights, haematological parameters, and microscopic appearance of the organs from the treatment groups. Thus, the extract with these doses would not cause any acute or subchronic toxicity and organ damages in rats. The oral median lethal dose (LD_50_) might be more than 5000 mg/kg body weight [84,85,86].

The *O. stamineus* aqueous extract (0, 250, 500, 1000, and 2000 mg/kg/day) did not change pregnancy body weight gain, food and water consumption, and caused no other sign of maternal toxicity in pregnant rats on gestation days 6–20. There was no embryo lethality and prenatal growth retardation either [87].

The genotoxicity of *O. stamineus* aqueous extract was evaluated by the *Salmonella*/microsome mutation assay and the mouse bone marrow micronucleus test. The result showed that *O. stamineus* extract was not toxic to *Salmonella* strains and did not have any potential to induce gene mutations in *Salmonella* strains. The aqueous extract was also not toxic to the mouse bone marrow. Thus, the use of *O. stamineus* aqueous extract had no genotoxic risk [88].

## 3. Clinical Applications

The medical plant *O. stamineus* has been used in the treatment of some kidney diseases and to improve the renal function for many years in clinical in China, including diabetic nephropathy, chronic nephritis, chronic renal failure, etc. [89].

In a clinical study, the effective rate of the prescription of *Cordyceps sinensis* and *O. stamineus* on diabetic nephropathy was 76.7% among 30 patients. The prescription could decrease the levels of fasting and postprandial blood glucose, glycosylated hemoglobin (HbA1c), urinary protein and serum creatinine, and increase endogenous creatinine clearance rate [90]. In another clinical study, the effective rate of the capsule of *Cordyceps sinensis* and *O. stamineus* on diabetic nephropathy was 83.3% among 30 patients. The capsule could decrease the levels of urine protein, serum creatinine, and urea nitrogen. *O. stamineus* might have a good effect on diabetic nephropathy by improving the function of renal function [91]. The Chongcaoshencha capsules used in the literature were prepared by Heilongjiang University of Chinese Medicine, including 1 g *Cordyceps sinensis*, 40 g raw *Astragalus membranaceus*, 2 g leeches, 10 g rhubarb, 15 g *Alpinia katsumadai,* and 20 g *O. stamineus*. Each capsule was 0.45 g [92,93].

## 4. The Pharmacodynamic Material Basis

### 4.1. Phenolic Acids

**Figure 2:**
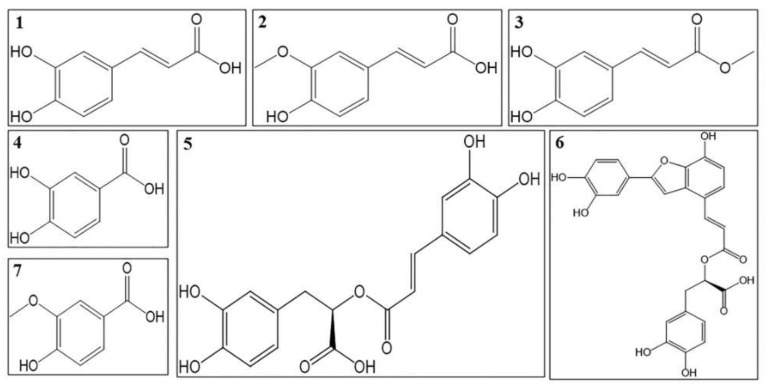
Structures of phenolic acids with antidiabetic effects; (**1**) Caffeic acid, (**2**) Ferulic acid, (**3**) Methyl caffeate, (**4**) Protocatechuic acid, (**5**) Rosmarinic acid, (**6**) Salvianolic acid and (**7**) Vanillic acid.

There are almost 50 phenolic acids and their derivatives isolated from *O. stamineus* up to now. The structures of antidiabetic phenolic acids are summarized in Figure 2 and the mechanisms of these compounds are summarized in Table 2. Ferulic acid, methyl caffeate, vanillic acid, protocatechuic acid and rosmarinic acid lower blood glucose level in vivo [94,95,96]. Salvianolic acid C and rosmarinic acid had been proved to have inhibitory activity on α-glucosidase [97,98]. Vanillic acid and rosmarinic acid are both antioxidants [94,99]. Rosmarinic acid also have anti-inflammatory activity, which reduce NO production and the levels of pro-inflammatory cytokines such as TNF-α, IL-1β, IL-6 [100,101]. Protocatechuic acid and rosmarinic acid regulate the lipid metabolism in diabetic animals. Protocatechuic acid lowers TC, TG, LDL-C levels and increases HDL-C level [102,103]. Methyl caffeate increases hepatic glycogen level and reduces gluconeogenesis through lowering glucose-6-phosphatase activity. It also increases glucose uptake by higher GLUT4 expression [96]. Rosmarinic acid also increases the glucose uptake of muscle cells through activation of adenosine 5′-monophosphate-activated protein kinase (AMPK) phosphorylation and glucose transporter-4 (GLUT4) expression. It promotes insulin secretion and improves insulin resistance by inhibiting dipeptidyl peptidase-4 (DPP-4) and PTB1B [104]. DPP-4 is an enzyme which can cleave the peptide bond in GLP-1 and result in a very low affinity between GLP-1 and the receptors. Thus, inhibition of DPP-4 can increase GLP-1 level and lower blood glucose level [105]. Methyl caffeate and rosmarinic acid could protect islet β-cells [96]. Ferulic acid and rosmarinic acid also have anti-glycation effects to decrease the formation of AGEs [95,106].

For diabetic complications, vanillic acid ameliorated diabetic liver dysfunction by lowering the levels of aspartate aminotransferase (AST) and alanine aminotransferase (ALT). It also decreases the levels of urea, uric acid, and creatinine in kidney [106]. Protocatechuic acid and rosmarinic acid reduce histological changes in kidney tissues in diabetic nephropathy animals [103,107]. Ferulic acid and protocatechuic acid increase the activity of SOD in cardiac tissues and decrease cardiomyocytes apoptosis to treat diabetic cardiomyopathy [95,108]. For diabetic retinopathy, lithospermic acid B improves oxidative stress in retinal tissues, prevents vascular leakage and basement membrane thickening in retinal capillaries [109].

### 4.2. Flavonoids

**Figure 3:**
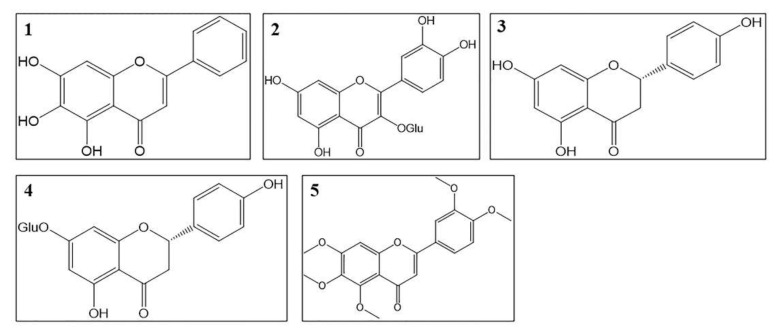
Structures of flavonoids with antidiabetic effects; (**1**) Baicalein, (**2**) Isoquercitrin, (**3**) Naringenin, (**4**) Prunin and (**5**) Sinensetin.

To date, more than 20 flavonoids have been isolated from *O. stamineus*. Most of them are flavones, especially polymethoxy substituted flavones. The structures of antidiabetic flavonoids are summarized in Figure 3 and the mechanisms of these compounds are summarized in Table 3. Isoquercitrin, baicalein, and naringenin lower blood glucose level in vivo. They also increase SOD activity, lower MDA level, and regulate lipid metabolism [112,113,114]. Sinensetin and prunin have inhibitory activity on α-glucosidase [68,115]. Prunin improves insulin resistance through inhibitory activity against PTP1B and the expression of Akt and PI3K [115]. Isoquercitrin and baicalein increase mRNA expression of IR, Akt, and PI3K to enhance insulin sensitivity [113,116]. Prunin and isoquercitrin increase glucose consumption of hepatocytes [115,116]. Baicalein promotes glucose uptake and glycolysis by inhibiting the expression of glucose-6-phosphatase, and inhibits gluconeogenesis of hepatocytes [112]. Naringenin increases the expression of GLUT-4 to promote glucose uptake [117,118]. Besides, isoquercitrin lowers DPP-IV mRNA levels and increases GLP-1 levels. Isoquercitrin and naringenin protected pancreatic tissues in a histopathological study and improved pancreatic necrosis [116,119].

In diabetic liver dysfunction, isoquercitrin and naringenin reduces serum ALT and AST levels, prevent hepatic apoptosis, and promote the regeneration of hepatocytes [116,120]. Baicalein and naringenin ameliorate diabetic nephropathy by mitigating renal oxidative stress, normalizing serum pro-inflammatory cytokines levels, improving structural changes in renal tissues, and reducing apoptosis [120,121,122,123]. Besides, naringenin might also ameliorate diabetic vascular dysfunction, diabetic neuropathy, and diabetic retinopathy [124,125,126].

### 4.3. Triterpenoids

**Figure 4:**
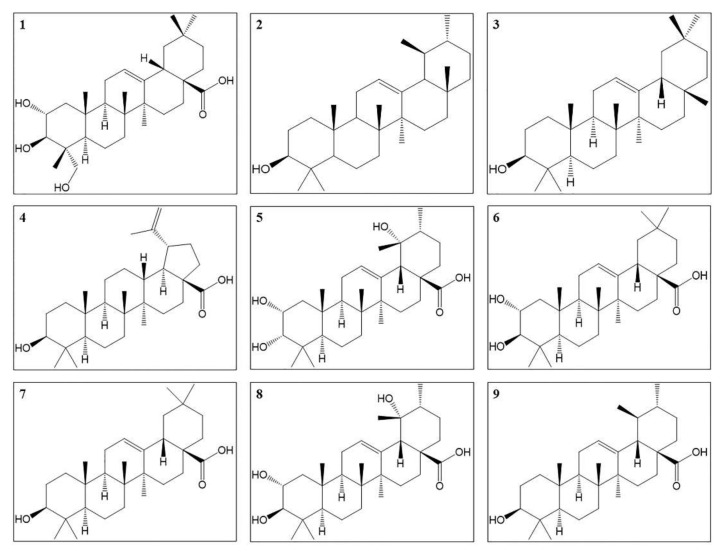
Structures of triterpenoids with antidiabetic effects; (**1**) Arjunolic acid, (**2**) α-Amyrin, (**3**) β-Amyrin, (**4**) Betulinic acid, (**5**) Euscaphic acid, (**6**) Maslinic acid, (**7**) Oleanolic acid, (**8**) Tormentic acid and (**9**) Ursolic acid.

There are almost 20 triterpenoids isolated from *O. stamineus*. The structures of antidiabetic triterpenoids are summarized in Figure 4 and the mechanisms of these compounds are summarized in Table 4. α, β-Amyrin, arjunolic acid, betulinic acid, tormentic acid, oleanolic acid, and ursolic acid lower blood glucose level in vivo. Among them, oleanolic acid and ursolic acid have an inhibitory activity on α-glucosidase [130,131]. Arjunolic acid, oleanolic acid, and ursolic acid have antioxidant activities to scavenge free radicals, while oleanolic acid and ursolic acid also have anti-inflammatory activities [100,132,133,134]. α, β-Amyrin, arjunolic acid, tormentic acid, oleanolic acid, and ursolic acid lower the levels of TC, TG, LDL-C and leptin, increase serum HDL-C level to regulate the lipid metabolism [133,135,136,137,138]. Maslinic acid, oleanolic acid, and ursolic acid improve insulin resistance and enhance insulin sensitivity respectively by a higher expression of IR, IRS, Akt, and PIP1B inhibitory activity [132,133,139]. Tormentic acid promotes glucose uptake by increasing the levels of phospho-AMPK and GLUT4 in skeletal muscle [136]. Oleanolic acid inhibits gluconeogenesis by decreasing expression of glucose-6-phosphatase [133]. Maslinic acid and ursolic acid increase the hepatic glycogen accumulation [135,139]. α, β-Amyrin, arjunolic acid, and betulinic acid protect islet cells and decrease cell death [138,140,141]. Oleanolic acid has anti-glycation effects to inhibit the formation of AGEs products [142].

In diabetic liver dysfunction, arjunolic acid reduces the secretion of ALT and the overproduction of ROS and RNS [141]. While oleanolic acid decreases ROS production, NF-κB expression and IL-1β, IL-6 and TNF-α levels in liver, and increases the activity of SOD [133]. Arjunolic acid and tormentic acid both reduce histological changes in liver tissues [136,141]. With regard to diabetic nephropathy, arjunolic acid, ursolic acid, and betulinic acid improve the lesions of renal tissues [143]. Maslinic acid, ursolic acid, and oleanolic acid decrease ROS and MDA levels and increase SOD activity in renal tissues [133,144,145]. Arjunolic acid, ursolic acid, and betulinic acid reduce the ratio of kidney weight to body weight, the levels of blood urea nitrogen (BUN), and creatinine. Ursolic acid also lowers urine albumin excretion [141,146,147]. Maslinic acid also increases Na^+^ excretion rate and glomerular filtration rate, and decreases creatinine level [145,148]. For diabetic cardiomyopathy, ursolic acid decreases the levels of AGEs, TNF-α, IL-1β, and ROS, increases the activity of SOD in myocardium [149]. Arjunolic acid reduces histological changes in cardiac tissues and reduces the number of apoptotic cells [137].

## 5. Discussion

**Figure 5:**
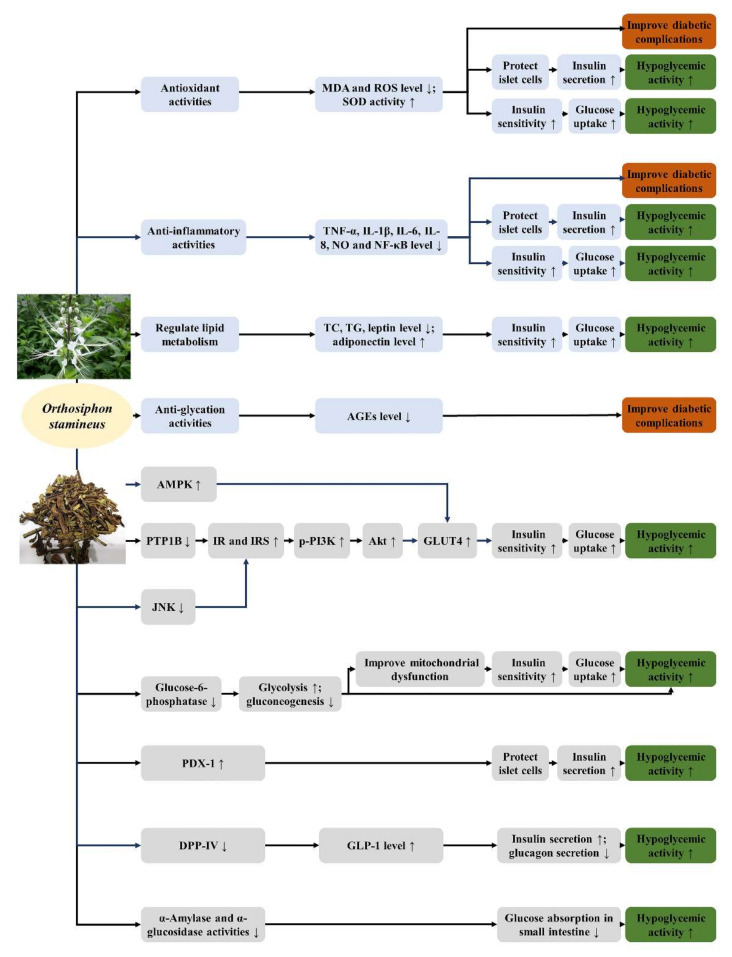
The mechanisms of *O. stamineus* in the treatment of diabetes and its complications. (The blue part shows other activities related to antidiabetic activity. The grey part is the pathways, targets, and enzymes related to antidiabetic activity).

**Figure 6:**
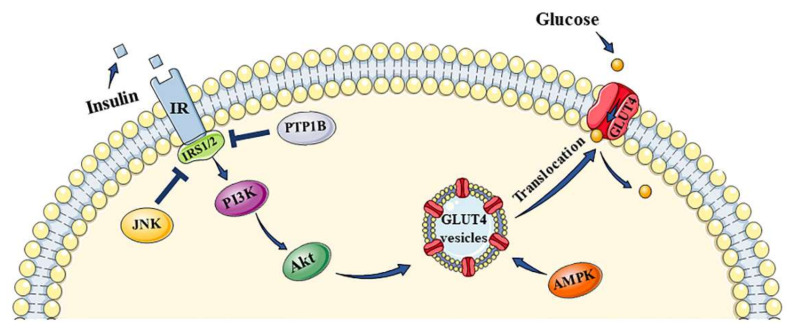
Summary of the PI3K/Akt, AMPK, JNK signal pathways related to insulin resistance. Arrows indicate activation, thick lines indicate inhibition.

*O. stamineus* is a potential natural product to treat diabetes and its complications. The mechanisms of *O. stamineus* in the treatment of diabetes and its complications are summarized in Figure 5. The antioxidant activity, anti-inflammatory activity, anti-glycation activity and lipid metabolism regulation are all related to antidiabetic activity. *O. stamineus* protects the islet cells, enhances insulin sensitivity, and improves diabetic complications by lowering the levels of free radicals and inflammatory factors. It also improves insulin resistance by lowering the levels of free fatty acids and leptin. The lower level of AGEs is able to improve diabetic complications. Besides, *O. stamineus* enhances insulin sensitivity and improves insulin resistance through other pathways, such as the PI3k/Akt signaling pathway, the AMPK pathway, and the JNK pathway (summarized in Figure 6) [153,154,155]. The PTP1B activity might also be related to the PI3k/Akt pathway. Some diterpenes isolated from *O. stamineus* had inhibitory activity on PTP1B. The hexane fraction of 70% ethanol extract and some flavonoids (prunin, isoquercitrin, baicalein) can increase the expression of PI3K and Akt. Rosmarinic acid and tormentic acid could increase the expression phospho-AMPK. Arjunolic acid and ursolic acid prevent the expression of JNK. In addition, *O. stamineus* reduces glucose absorption from the small intestine by inhibiting the activities of α-amylase and α-glucosidase, promotes insulin secretion by elevating PDX-1 level, and lowers GLP-1 level. It could also promote glycolysis and inhibit gluconeogenesis by inhibiting glucose-6-phosphatase. However, some current experiments have only studied the antidiabetic effects and results of *O. stamineus*, such as reducing blood glucose level, improving insulin level, increasing glucose uptake, and reducing glucose absorption, without further exploration of its mechanisms and pathways. The relationship between *O. stamineus* extracts and AMPK, JNK pathways should be further studied.

Until now, investigations on the antidiabetic effects and mechanisms of *O. stamineus* have concentrated mainly on the effects of extracts, especially 50% ethanol extract and aqueous extract. The effects of extracts might be different because the levels of some metabolites vary in the plant from different places. Through literature research, it was seen that phenolic acids, flavonoids, and triterpenoids might be the main active components to treat diabetes and complications. To identify the major bioactive compounds responsible for antidiabetic effects, bioassay-guided isolation should be used. The mechanisms of pure compounds are also required to study, and there might be synergistic effects between these constituents.

In China and some southeastern Asian countries, *O. stamineus* has been used as traditional medicine for the treatment of diabetes and some kidney diseases for a long time. In recent years, by means of modern science and techniques, there have been more and more investigations in the mechanisms of *O. stamineus* in the treatment of diabetes and diabetic complications. However, most experiments are in vitro or using experimental animal models in vivo, which may be different from the effects and mechanisms of *O. stamineus* in the human body. In addition, clinical research is very limited. *O. stamineus* was only used to treat chronic renal diseases in clinical, such as chronic glomerulonephritis [156]. But because *O. stamineus* might be a good antidiabetic candidate to reduce blood glucose levels and alleviate kidney injury, it could also be designed to study the clinical treatment of diabetic nephropathy in the future.

At present, diabetes is treated with oral hypoglycemic drugs and insulin injections. The glucose-lowering drugs include α-glucosidase inhibitors (acarbose, miglitol), insulin sensitizers (metformin, thiazolidinediones, biguanides), insulin secretagogues (sulfonylureas), etc. However, most of these medications may have side-effects, including hypoglycemia, weight gain, liver damage, gastrointestinal disturbance, lactic acidosis, edema, headache, dizziness, anemia, nausea, and even death. Besides, long-term use of insulin may decrease insulin receptor sensitivity, resulting in insulin resistance [157]. In the future, glucose-lowering drugs might be combined with *O. stamineus* to find out if they can reduce these side-effects and increase antidiabetic effects. Besides, some other natural products with antidiabetic activities can also be used with *O. stamineus* to test the combined effects in the treatment of diabetes and diabetic complications. *Cordyceps sinensis*, *Astragalus membranaceus*, *Rheum officinale*, and leech have been combined with *O. stamineus* to treat diabetic nephropathy as a Chinese traditional medicine prescription [91].

## 6. Methods

This review was performed and reported according to PRISMA guidelines [37,38]. The flowchart of selected articles is shown in Figure 1.

### 6.1. Search Strategy

Three databases (ScienceDirect, PubMed and Web of Science) were used to search relevant articles using the terms “((*Clerodendranthus spicatus*) OR (*Orthosiphon stamineus*) OR (*Orthosiphon aristatus*)) AND ((diabetes) OR (antidiabetic) OR (hypoglycemic) OR (diabetic complications))”. No time restriction was used. The initial search included 281 articles. The results of ScienceDirect, PubMed, and Web of Science were respectively exported as RIS, NBIB, and ISI files. All obtained files were then imported into EndNote X9 to generate a library.

### 6.2. Eligibility Criteria

The research included in this review met the following criteria: 1. the study reported hypoglycemic activity or the treatment of diabetes and its complications of *O. stamineus* extract or its isolated compounds, 2. the study reported other biological activities related to diabetes treatment, such as antioxidant and anti-inflammatory activities, of *O. stamineus* extract or its isolated compounds, 3. the study reported the toxicity of *O. stamineus* extract or its isolated compounds.

The exclusion criteria of this review were as follows: 1. reviews, book chapters, patents, meeting papers, 2. non-English language papers, 3. lack of access to the full-text of the paper, 4. no relevance to the plant *O. stamineus* or the field of diabetes and its complications. Besides, duplicate articles were also removed.

### 6.3. Data Extraction

Thirty-one studies met the criteria, and the data were extracted into Microsoft Excel 2007 sheet and inserted to Table 1. The information gathered from the studies included: 1. name of the first author, 2. publication year, 3. the tested substance, 4. the study design and protocol, 5. main results.

## 7. Conclusions

In conclusion, *O. stamineus* is a potential agent to treat diabetes and diabetic complications. The extracts of *O. stamineus*, including 50% ethanol extract, chloroform extract, aqueous extract, and hexane extract, could be used to treat diabetes through mechanisms including inhibiting the activities of α-amylase and α-glucosidase, antioxidant and anti-inflammatory activities, regulating lipid metabolism, promoting insulin secretion, ameliorating insulin resistance, enhancing insulin sensitivity, increasing glucose uptake, promoting glycolysis, inhibiting gluconeogenesis, promoting the secretion of GLP-1, and antiglycation effects. The mechanisms of insulin resistance might also be related to the PI3k/Akt signaling pathway, the AMPK pathway, and the JNK pathway. The aqueous extract could also be used for diabetic nephropathy treatment. Besides, some main active components, such as rosmarinic acid, ferulic acid, methyl caffeate, vanillic acid, protocatechuic acid, isoquercitrin, baicalein, naringenin, arjunolic acid, betulinic acid, tormentic acid, oleanolic acid, ursolic acid, maslinic acid, siphonols B, orthosiphols B, G, I, and N also had good effects in the treatment of diabetes and its complications. However, it needs further study on pharmacodynamic substance basis and the mechanisms of effective constituents.

## Figures and Tables

**Table 1:** Summary of articles reported for antidiabetic effects and toxicity of *O. stamineus*.

No.	Tested Substances	Study Design and Protocol	Ref.
1	2-Caffeoyl-L-tartaric acid, rosmarinic acid	α-Glucosidase inhibitory activity and molecular docking	[66]
2	95% EEF of 80% ethanol extract	Oral glucose tolerance test in normal C57BL/6J mice	[39]
		α-Glucosidase inhibitory activity	
3	Ethanol extract	α-Glucosidase and α-amylase inhibitory activity	[46]
4	Ethanol extract, aqueous and EtOAc fractions of ethanol extract, 25 compounds isolated from EtOAc fraction	Measurement of pro-inflammation cytokine in vitro	[56]
		Xylene-induced acute inflammatory model of mice	
5	Ethanol extract	α-Glucosidase inhibitory activity	[46]
		Antioxidant activity (DPPH and FRAP assays)	
6	Siphonol B, orthosiphols B, G, I and N	Measurement of 2-NBDG uptake in 3T3-L1 adipocytes	[74]
		PTP1B inhibitory activity	
7	Aqueous extract	Oral glucose tolerance test	[62]
		Plasma analysis (insulin, cholesterol, GLP-1, and ghrelin levels) in diabetic rats	
8	70% Ethanol extract and 9 fractions	Antioxidant activity (DPPH assay)	[47]
9	50% Methanol extract	Antioxidant activity (DPPH, ABTS, iron chelating and FRAP assays)	[49]
10	Ethanol extract	Pancreatic lipase inhibitory activity in vitro	[40]
		Biochemical serum analysis (TG, TC, LDL, lipase, and glucose levels) in HFD-induced rats	
		Measurement of leptin, adiponectin, insulin, and HOMA-IR index in HFD-induced rats	
		Determination of antioxidant activity in liver tissue in HFD-induced rats	
		Histological assessment of liver tissues in HFD-induced rats	
11	Aqueous extract	Antioxidant activity (DPPH and ABTS assays)	[50]
		Cytotoxicity assay, embryotoxicity assay	
12	Aqueous extract	^1^H-NMR spectroscopic analysis of urine of diabetic rats	[63]
13	Aqueous, 50% ethanol and ethanol extracts	Acute toxicity study in rats	[84]
14	Clerodens A–D	Assay for inhibitory ability against LPS-induced NO production in RAW264.7 macrophages	[16]
15	50% Ethanol extract	Oral carbohydrate challenge tests in normal and diabetic rats (respectively starch, surcose, and glucose loading)	[41]
16	Hexane fraction of 70% ethanol extract	Glucose stimulated insulin secretion test	[75]
		Real time-polymerase chain reaction	
17	Aqueous extract	Effects on glucose uptake	[78]
18	Aqueous extract	The developmental toxicity study in pregnant rats	[87]
19	Sub-fraction 2 of chloroform extract	Determination of blood glucose level in diabetic rats	[34]
		Measurement of glucose absorption in the everted rat jejunum, measurement of glucose uptake in isolated rat hemi-diaphragms	
20	Methanol extract	Antioxidant activity (DPPH assay)	[48]
21	Aqueous extract	Effects on glucose uptake and glucose consumption	[77]
22	50% Ethanol extract and sinensetin	α-Glucosidase and α-amylase inhibitory activity	[68]
23	Aqueous extract	Antioxidant activity (ABTS and FRAP assays)	[51]
		Determination of anti-AGEs formation capacity	
24	Aqueous extract	*Salmonella*/microsome mutation assay, mouse bone marrow micronucleus test	[88]
25	Chloroform extract and its sub-fraction 2	Subcutaneous glucose tolerance test in normal rats	[42]
26	50% Ethanol extract	Acute toxicity study in rats	[86]
		Subchronic toxicity study in rats	
27	Methanol extract	Acute toxicity study in rats	[85]
28	Aqueous extract	Oral glucose tolerance test and plasma analysis in normal and diabetic rats	[43]
29	Aqueous, 50% methanol, methanol, 70% acetone and chloroform extracts	Antioxidant activity (DPPH assay)	[52]
30	Neoorthosiphonone A	Assay for inhibitory ability against LPS-induced NO production in macrophage-like J774.1 cells	[57]
31	Siphonols A–E	Assay for inhibitory ability against LPS-induced NO production in macrophage-like J774.1 cells	[58]

**Table 2:** The effects and mechanisms of some phenolic acids in *O. stamineus* in the treatment of diabetes and diabetic complications.

No.	Compounds	Diabetes and Diabetic Complications	Effects and Mechanisms	Ref.
1	Caffeic acid	Diabetes	Lowers blood glucose level	[110]
2	Ferulic acid	Diabetes	Lowers blood glucose level; lowers the activities of ALT and AST in the serum	[95]
		Diabetic cardiomyopathy and liver dysfunction	Decreases the content of AGEs in the liver and heart; decreases the number of apoptotic hepatocytes and cardiomyocytes; reduces histological changes in liver tissues; increases the activity of SOD in the liver and heart	
3	Methyl caffeate	Diabetes	Lowers blood glucose level; increases hepatic glycogen level; decreases glucose-6-phosphatase activity; increases the size and number of islets; increases GLUT4 expression; improves β-cells	[96]
4	Protocatechuic acid	Diabetes	Lowers blood glucose level	[102,103]
		Diabetic nephropathy and liver dysfunction	Decreases lipid hydroperoxides in liver and kidney; decreases TC, TGs, LDL-C and VLDL-C levels and increases HDL-C level in liver and kidney; reduces histological changes in liver and kidney	
5	Rosmarinic acid	Diabetes	Reduces blood glucose, TC, TGs and lipid peroxides levels; inhibitors of α-amylase, α-glucosidase, DPP-IV and PTB1B; lowers the formation of MDA and AGEs; reduces the levels of pro-inflammatory cytokines such as TNF-α, IL-1β, IL-6, NO and nuclear factor kappa-B (NF-κB); increases the activity of SOD; increases the glucose uptake of muscle cells through activation of AMPK phosphorylation; improves insulin sensitivity; increases GLUT4 expression in skeletal muscle; protects pancreatic β-cells	[98,99,100,101,104,106]
		Diabetic vascular dysfunction	Decreases IL-1β and TNF-αlevels and the expression of endothelin converting enzyme-1; improves structural alterations in the endothelium	[111]
6	Salvianolic acid C	Diabetic cardiomyopathy	Enhances intracellular adenosine triphosphate (ATP) content in the myocardial tissues; reduces ROS, lipid peroxidation and protein carbonylation level in myocardial tissues; improves SOD level in cardiac tissues; reduces histological abnormality	[108]
7	Vanillic acid	Diabetes	Lowers blood glucose level; decreases the concentration of lipid hydroperoxides	[94]
		Diabetic nephropathy and liver dysfunction	Increases the activities of antioxidants in kidney and liver; reduces the levels of AST and ALT in liver; decreases the levels of urea, uric acid, and creatinine in kidney; reduces histological changes in liver and renal tissues	

**Table 3:** The effects and mechanisms of some flavonoids in *O. stamineus* in the treatment of diabetes and diabetic complications.

No.	Compounds	Diabetes and Diabetic Complications	Effects and Mechanisms	Ref.
1	Baicalein	Diabetes	Lowers blood glucose and MDA level; inhibits gluconeogenesis of hepatocytes; decreases the expressions of glucose-6-phosphatase; increase SOD activity; promotes glucose uptake and glycolysis; increases the expression of PI3K and Akt; increase hepatic glycogen level	[112,113,127,128]
		Diabetic nephropathy	Lowers HOMA-IR level; restores normal renal function; mitigates renal oxidative stress; lowers the level of NF-κB; ameliorates the structural changes in renal tissues; normalizes the levels of serum pro-inflammatory cytokines and liver function enzymes	[122]
2	Isoquercitrin	Diabetes	Lowers blood glucose, serum HOMA-IR, DPP-IV mRNA levels; increases glucose uptake of hepatocytes; increases mRNA expression of Akt and PI3K; increases SOD, HDL-C, insulin and GLP-1 levels; improves pancreatic atrophy and necrosis	[116]
		Diabetic liver dysfunction	Reduces serum ALT and AST levels; prevents hepatocytes architecture and hepatic necrosis; suppresses apoptosis and promotes regeneration of hepatocytes	
3	Naringenin	Diabetes	Lowers blood glucose, MDA and glycosylated hemoglobin levels; lowers the activities of ALT and AST in serum; increases serum insulin levels; increases the expression of GLUT-4; protects the pancreatic tissues in histopathological study; normalizes lipid concentrations in the serum	[114,117,118,119]
		Diabetic liver dysfunction	Decreases lipid peroxidation level in liver; decreases the number of vacuolated liver cells and degree of vacuolisation	[120]
		Diabetic nephropathy	Decreases the 24 h-urinary protein, kidney index and glomerular area; increases creatinine clearance rate; decreases lipid peroxidation level in kidney tissue; increases the activity of SOD; decreases renal IL-1β, IL-6 and TNF-α levels; lowers NF-κB p65 expression in kidney; improves kidney histology; reduces apoptosis	[120,121,123,129]
		Diabetic retinopathy	Increases levels of neuroprotective factors, tropomyosin related kinase B and synaptophysin in diabetic retina; ameliorates the levels of apoptosis regulatory proteins in diabetic retina	[126]
4	Prunin	Diabetes	Inhibitory activity against PTP1B and α-glucosidase; stimulates glucose uptake; increases the expression of p-Akt and p-PI3K	[115]
5	Sinensetin	Diabetes	Inhibitory activity on α-glucosidase and α-amylase	[68]

**Table 4:** The effects and mechanisms of some triterpenoids in *O. stamineus* in the treatment of diabetes and diabetic complications.

No.	Compounds	Diabetes and Diabetic Complications	Effects and Mechanisms	Ref.
1	Arjunolic acid	Diabetes	Lowers blood glucose, NO, MDA and protein carbonylation levels; increases the activities of antioxidant enzymes; increases cell viability and decreases cell death; reduces pathological lesion; prevents the expression of c-Jun N-terminal kinase (JNK)	[134,137,141]
		Diabetic cardiomyopathy	Reduces the levels of vascular inflammation markers; increases the activities of the antioxidant enzymes and cellular redox ratio; decreases DNA oxidation in cardiac tissue; reduces histological changes in cardiac tissues; reduces the number of apoptotic cells	
		Diabetic liver dysfunction	Reduces the secretion of ALT, the overproduction of ROS and RNS; reduces histological changes in liver tissues; prevents cell death	
		Diabetic nephropathy	Reduces kidney weight to body weight ratio, glomerular area, glomerular volume, BUN and creatinine; reduces the activation of NF-κB; prevents cell death; keeps the kidney close to normal physiological state	
2–3	α, β-Amyrin	Diabetes	Lowers blood glucose, LDL, VLDL levels; increases insulin levels; protects islets of Langerhans	[138]
4	Betulinic acid	Diabetes	Lowers blood glucose level; improves insulin sensitivity; decreases insulin resistance by the alternation of some insulin biomakers; improves pancreatic islets diameter and number; improves pancreatic histology	[140]
5	Euscaphic acid	Diabetes	Inhibitory activity on α-glucosidase and the formation of Amadori, which is an early product of nonenzymatic glycosylation	[150]
6	Maslinic acid	Diabetes	Increases hepatic glycogen accumulation; inhibits glycogen phosphorylase activity; induces the phosphorylation level of IRβ and Akt	[139]
		Diabetic nephropathy	Increases the activity of antioxidant enzymes in renal tissues; increases Na^+^ output, Na^+^ excretion rates, fractional excretion of Na^+^; increases glomerular filtration rate; decreases plasma aldosterone and creatinine levels; diminishes the expression of GLUT1 and GLUT2 in diabetic kidney	[145,148]
7	Oleanolic acid	Diabetes	Lowers blood glucose, LDL and free fatty acids levels; increases insulin level; inhibitory activity on α-glucosidase, α-amylase and PIP1B; inhibits the formation of AGEs products; improve insulin tolerance; inhibits gluconeogenesis; increases serum HDL level; decreases levels of IL-1b, IL-6 and TNFα; increases the activity of SOD; improve glycogen level by the increasing expression of Akt and decreasing expression of glucose-6-phosphatase; increases the expression of IR and IRS-1	[131,133,142,151]
		Diabetic liver dysfunction	Decreases the levels of IL-1β, IL-6 and TNFα in liver; decreases the expression of NF-κB; decreases ROS production; increases the activity of SOD	[133,152]
8	Tormentic acid	Diabetes	Lowers blood glucose, leptin and total lipids levels; increases the protein contents of phospho-AMPK and GLUT4 in skeletal muscle	[136]
		Diabetic liver dysfunction	Reduces histological changes in liver tissues; decreases the mRNA level of glucose-6-phosphatase in liver tissues; increases the protein contents of hepatic phospho-AMPK	
9	Ursolic acid	Diabetes	Lowers blood glucose, MDA and LDL levels; inhibits α-amylase and α-glucosidase activity; increases SOD activities; decreases TNF-α and IL-1β level; increases liver glycogen level; decreases the expression of PTP-1B protein; increases the expression of IRS-2 protein	[130,132,135]
		Diabetic cardiomyopathy	Decreases levels of AGEs, TNF-α, IL-1β and ROS; increases the activity of SOD in myocardium	[149]
		Diabetic nephropathy	Lowers the levels of BUN, creatinine and MDA; lowers urine albumin excretion, renal oxidative stress level, NF-κB activity; prevents the expression of JNK; improves renal structural abnormalities	[144,146,147]

## Data Availability

Not applicable.
